# The Impact of Chiropractic Care on Opioid Prescriptions in Veterans Health Administration Patients Receiving Low Back Pain Care

**DOI:** 10.1007/s11606-025-09556-w

**Published:** 2025-05-20

**Authors:** Anthony J. Lisi, Lori A. Bastian, Cynthia A. Brandt, Brian C. Coleman, Brenda Fenton, Joseph T. King, Joseph L. Goulet

**Affiliations:** 1 Pain Research, Informatics, Multimorbidities, and Education (PRIME) Center, VA Connecticut Healthcare System, West Haven, CT USA; 2 Yale School of Medicine, New Haven, CT USA; 3 Section of Neurosurgery, VA Connecticut Healthcare System, West Haven, CT USA

**Keywords:** Veterans health, chiropractic, analgesics, opioid, low back pain, primary healthcare

## Abstract

**Background:**

Key nonpharmacologic therapies, including those routinely provided by chiropractors, are recommended first-line treatments for low back pain (LBP). Little is known on whether such care provided in the Veterans Health Administration (VA) has a downstream effect on the use of other healthcare services, including opioid prescriptions.

**Objective:**

To evaluate the impact of chiropractic care on receipt of opioid prescriptions within 365 days of an incident primary care provider (PCP) visit for LBP among opioid-naïve VA patients.

**Design:**

Cross-sectional analysis with longitudinal follow-up.

**Participants:**

Patients had an LBP visit with a VA PCP between 10/1/2015 and 9/30/2020, without any VA LBP visit in the preceding 18 months, and then 2 subsequent VA LBP visits in the following 12 months.

**Main Measures:**

VA electronic health record data including outpatient visits, prescriptions, and comorbid diagnoses.

**Key Results:**

A total of 128,377 patients met study criteria. The hazard ratio for opioid prescription in a propensity-matched sample was 0.77 (95% CI 0.71–0.83), indicating a significantly lower risk for receipt of an opioid prescription among chiropractic care users in the 365-day follow-up adjusting for potential confounders. The cumulative incidence of opioid prescriptions was 13.0% for chiropractic care users and 16.8% for non-users and the number needed to treat was 27.

**Conclusions:**

The results of this study show that nonpharmacologic chiropractic care can be an important component of opioid sparing strategies for VHA patients with LBP.

**Supplementary Information:**

The online version contains supplementary material available at 10.1007/s11606-025-09556-w.

## BACKGROUND

Since the start of its Opioid Safety Initiative (OSI) in October 2013^1^, through its current Pain Management Opioid Safety and Prescription Drug Monitoring Program (PMOP),^2^ the Veterans Health Administration (VA) has been working to embrace a culture of effective pain treatment while reducing the risks associated with long-term opioid therapy.^3^ VA’s strategy includes provider and patient education, risk mitigation, addiction treatment, and expansion of pain care treatment options, including nonpharmacologic therapies. One key consideration has been attentiveness that chronic pain rarely occurs in isolation but rather co-occurs with other medical, mental health and substance use disorders in veterans.^4^ These comorbidity patterns are crucial to consider as they can complicate the clinical presentation, course, as well as treatment options and outcomes for veterans. With respect to treatment options, in 2016, a VA Health Services Research and Development State of the Art Conference issued expert recommendations that a group of evidence-based nonpharmacologic therapies for chronic musculoskeletal pain should be available across the VA healthcare system.^5^ Of the pain conditions for which nonpharmacologic treatments may be indicated, perhaps none is more common and burdensome than low back pain (LBP). In North America, the age-standardized prevalence estimate for LBP is 10.5%, and rate of Years Lived with Disability is 1160 per 100,000.^6^ LBP disproportionately affects Veterans compared to the non-Veteran population.^7^ In VA patients, “M54.5, Low Back Pain” was the most common musculoskeletal diagnosis code in the first 2 years of ICD-10 adoption (October 1, 2015–September 30, 2017) both by outpatient visits (18.3%) and by unique patients (43.1%).^8^ It has been estimated that over 80% of incident visits for LBP conditions in VA occur in primary care provider (PCP) clinics.^9^

LBP clinical practice guidelines recommend evidence-based nonpharmacologic treatment options as first-line therapies including exercise, superficial heat, spinal manipulation, massage, cognitive behavioral therapy, and others.^10^ In the USA, one of the most common approaches for LBP patients is chiropractic care. In an assessment of commercial insurance and Medicare Advantage claims data, the top two entry points for new treatment episodes for LBP were PCPs (53.0%) and chiropractors (23.1%).^11^ VA began providing chiropractic care “on-station”—care delivered by VA providers in VA medical facilities—in 2004,^12^ and continues to expand access,^13^ yet its overall use remains very low compared to the US general population (4.1% in fiscal year 2023, compared to 11–14% in the US general population).^14–16^

Prior observational studies outside VA have shown that patients receiving chiropractic visits for LBP tend to subsequently use less of other healthcare services such as advanced imaging, therapeutic injections, and pharmacological therapies, including opioids.^11,17–19^ More attention has been given to the associations between chiropractic care receipt and medication use, including tramadol, gabapentin, and opioid use. A recent meta-analysis of six studies of adults with noncancer pain found chiropractic care users to have 64% lower odds of receipt of an opioid prescription than non-users (odds ratio = 0.36, 95% CI = (0.30, 0.43), *p* < 0.001; range of length of follow-up: 7 days to 1 year).^20^ Similarly, two recent Canadian studies^21,22^ found lower rates of opioid prescriptions in chiropractic care recipients where study participants were previously prescribed opioids. An earlier study in VA found the veterans of Operations Enduring Freedom/Iraqi Freedom/New Dawn (OEF/OIF/OND) who had at least one visit to a VA chiropractic clinic had lower rates of receipt of an opioid prescription in the 90 days after first chiropractic visit compared to the 90 days before the visit.^23^ Opioid prescription was higher among those veterans with moderate-severe pain, diagnosis of PTSD or depression and current smoking. However, these studies all considered samples inclusive of patients with previous and active use of prescription opioids, leaving open the question of the effect of chiropractic care on prescription opioid use in an opioid-naïve population.

The purpose of this study is to examine the longitudinal relationship between receipt of chiropractic care and receipt of opioid prescriptions within 1 year of follow-up after an incident PCP visit for LBP among opioid-naive patients receiving VA care for LBP.

## METHODS

### Study Design, Setting, Participants, and Data Sources

This study was a cross-sectional analysis with longitudinal follow-up of national VA Electronic Health Record (EHR) data from on-station VA care (provided on-site at VA medical facilities). Data were extracted from VA’s Corporate Data Warehouse consistent with previously established methods.^23,24^ We used the Strengthening the Reporting of Observational Studies in Epidemiology (STROBE) guidelines to report this observational study. The study was approved by the VA Connecticut Healthcare System Research and Development Committee ([1583217-25] Musculoskeletal Diagnoses Cohort: Examining Pain and Pain Care in the VA using Complementary and Integrative Health (CIH) Interventions (EXEMPT)).

### Patient Sample

**Figure 1:**
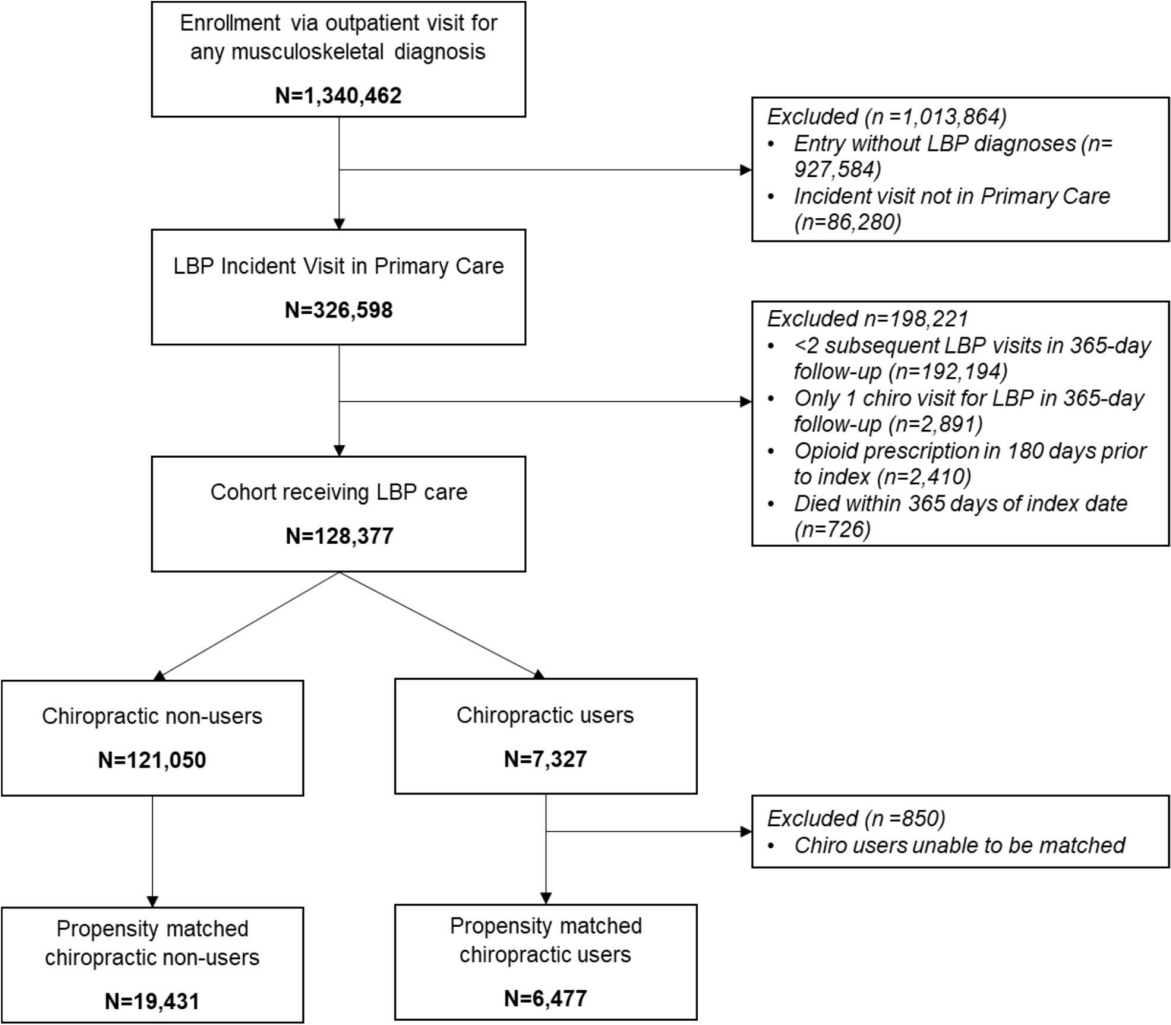
Flowchart for veteran inclusion for the examination of opioids in veterans in the year following a Primary Care encounter for incident LBP (total period: 10/1/2015–9/30/2021).

This patient sample was a subset of veterans with LBP from the larger group with any type of musculoskeletal disorder(s) (see Fig. 1). Patients were included in the study sample if they had a visit for LBP in a VA PCP clinic between 10/1/2015 and 9/30/2020. Our inclusion criteria maximized the likelihood of this sample index visit reflecting new initiation of healthcare for LBP by requiring no VA care for LBP in the 18 months preceding the incident visit. PCP clinics were defined based on VA clinic identifier “stop codes,” with a primary or secondary code of 301 (General Internal Medicine), 322 (Women’s Health), or 323 (Primary Care Medicine) linked to the visit.

We excluded patients with less than two subsequent on-station VA healthcare visits for LBP in any clinics within 365 days following their study index visit to limit the sample to those who were actively receiving care for LBP. This was done to minimize the likelihood of including cases that were self-limited to a single PCP visit, and/or cases where the LBP ICD- 10 code at the index visit may reflect a historical problem, rather than one the patient was currently experiencing. Additionally, patients who received an opioid prescription within 180 days prior to their study index were excluded. Patients who died within the 365 days after their study index date were also excluded.

### Exposure: Chiropractic Care

The primary exposure of interest was use of on-station VA chiropractic care for LBP. This was identified using the VA clinic stop code 436 (Chiropractic Care) linked to an outpatient visit. Only chiropractic care visits that included an LBP ICD- 10 diagnosis were included. “Chiropractic users” were defined as patients with a study index PCP visit and at least two subsequent LBP visits in a chiropractic clinic within 365 days. “Chiropractic non-users” were defined as patients with a study index PCP visit and at least two subsequent LBP visits in any clinics other than chiropractic within that timeframe.

### Outcome: Opioid Prescription

The primary outcome of this study was whether opioid prescriptions were filled (Yes/No) within 365 days of study index visit date, and the number of days from index visit to the receipt of an opioid prescription were for rate estimation. Given study inclusion criteria required a washout period of 180 days without an opioid prescription prior to study index, this outcome represents a “new” opioid prescription (opioid naïve for the purposes of this project). Any opioid prescription was included as the study outcome without restriction to count or dose. Receipt of opioid prescription may have included those for acute and/or chronic painful conditions.

### Covariables

Relevant covariables were included to adjust for potential confounding between the exposure and the outcome. Patient sociodemographic characteristics included sex of record, age, race/ethnicity, and marital status. Clinical characteristics were extracted from the EHR. Smoking status was obtained using structured clinical reminder data and text entries^25^ and categorized into “Never smoker”, “Current smoker”, “Former smoker”, and “Missing”. The patient’s index visit pain numerical rating scale (NRS) was extracted and categorized into “No pain (NRS = 0)”, “Mild pain (NRS = 1–3)”, “Moderate pain (NRS = 4–6)”, “Severe Pain (NRS = 7–10)”, or “Missing”.^26^

Comorbidities were identified based on the occurrence of ICD-10 diagnosis codes in the EHR within 12 months prior to and 6 months after the study index visit.^26^ These included co-occurring neck pain, major depressive disorder, generalized anxiety, post-traumatic stress disorder, traumatic brain injury, alcohol use disorder, and drug use disorder. An additional categorical variable was used to identify two groups of potentially higher-impact LBP diagnoses: serious/treatable diseases that may involve LBP (including cancers, fractures, and inflammatory arthritis) and lumbar radiculopathies. The list of ICD- 10 diagnoses used to define these groups is included as supplemental material. Additionally, two comorbidity indices were calculated based on ICD- 10 diagnosis groupings. The Charlson Comorbidity Index reflects poorer health status, mortality, and higher resource use.^27,28^ The Functional Comorbidity Index reflects patient physical function.^29,30^ Comorbidity indices were considered as continuous and categorical variables (0, 1, 2, 3 +). We assessed the use of nonpharmacologic treatments other than chiropractic care by identifying a group of five other clinics/services most likely to be delivering nonpharmacologic therapies for pain conditions: physical therapy, occupational therapy, mental health, complementary and integrative health (which includes most acupuncture visits), and health and well-being. We used VA Stop Codes to find visits to these clinics, and included only those with at least one LBP-related ICD-10 code.

### Statistical Analysis

The study’s analytic plan followed three cascading steps to calculate the hazard ratio for opioid prescription initiation within 365 days of the study index: (1) comparison of sociodemographic and clinical characteristics among chiropractic care users and non-users; (2) creation of propensity scores to reduce selection bias by controlling for a patient’s propensity to use chiropractic care (observed in Step #1) using inverse probability of treatment; and (3) calculation of a hazard ratio of opioid prescription for chiropractic care users vs. non-users, adjusting for the propensity score (as created in Step #2).

We used logistic regression and all covariables to characterize chiropractic care users vs. non-users. These results were used to calculate a propensity score for chiropractic care use to balance the characteristics in each group, matching chiropractic care users to non-users 1:3 on the propensity score (without replacement). We calculated the time to first opioid prescription from the date of the index LBP visit, or until the end of the 365-day observation period (censored) for all patients. We used Cox proportional hazards regression to estimate the hazard ratio for receipt of opioid prescription, including the propensity score as a covariable. All statistical analyses were conducted using SAS 9.4 (SAS Institute Inc.).

## RESULTS

### National Musculoskeletal Disorders Cohort

In our cohort we identified 128,377 veterans meeting study criteria including 7327 (5.71%) chiropractic care users and 121,050 (94.29%) non-users (Table 1). The chiropractic care user group were significantly younger (41.5 vs. 46.9 years), more likely to be women (15.6% vs. 12.9%), White (61.8% vs. 58.2%), and never married (20.4% vs. 17.4%) than non-users. Chiropractic care users were significantly more likely to have lumbar radiculopathy diagnoses (18.1% vs. 11.4%) and/or comorbid neck diagnoses (29.8% vs. 8.1%) (Table 2). Similarly, chiropractic care users were significantly more likely to have generalized anxiety (3.9% vs. 3.2%) or TBI (1.5% vs. 1.0%) diagnoses. In contrast, alcohol use disorders (4.7% vs. 5.3%), having ever smoked (56.5% vs. 60.0%), and average CCI score (0.50 vs. 0.89) were significantly lower in the chiropractic care users. The maximum NRS reported on the index date was significantly lower for the chiropractic care users. The groups were similar with respect to serious/treatable LBP diagnoses, comorbid major depression, PTSD, drug use disorders, and FCI scores. The use of other nonpharmacologic treatment modalities was lower among chiropractic care users (25.0% vs. 30.1%). The prevalence of receiving an opioid prescription on the LBP study index date was low overall and was significantly lower in the chiropractic care user group (1.3% vs. 2.0%) compared to the non-users.

**Table 1:** Sociodemographic Characteristics of Veteran Patients by Their Receipt of Chiropractic Care for LBP in the Year Following a Primary Care Visit for Incident Low Back Pain (*N* = 128,377)

Patient sociodemographic characteristics	Chiropractic care on-station		*p*-value
	Yes	No	
*N* (%)	7327 (5.71%)	121,050 (94.29%)	-
Sex (% female)	1147 (15.60%)	15,697 (12.90%)	< 0.0001
Age (continuous) (mean, SD)	41.49 (14.78)	46.94 (16.87)	< 0.0001
Age group			< 0.0001
Under 30	2183 (29.70%)	26,249 (21.60%)	
30–49	3127 (42.60%)	43,493 (35.90%)	
50 +	2017 (27.50%)	51,308 (42.30%)	
Race/ethnicity			< 0.0001
White	4534 (61.80%)	70,539 (58.20%)	
Black	1148 (15.60%)	23,812 (19.60%)	
Hispanic	891 (12.10%)	13,709 (11.30%)	
Other	754 (10.20%)	12,990 (10.70%)	
Marital status			< 0.0001
Missing/unknown	244 (3.33%)	3843 (3.17%)	
Divorced	1196 (16.30%)	21,465 (17.70%)	
Married	4064 (55.40%)	67,503 (55.70%)	
Never married	1499 (20.40%)	21,174 (17.40%)	
Separated	209 (2.80%)	4087 (3.30%)	
Single	20 (2.00%)	305 (2.00%)	
Widowed	95 (1.20%)	2673 (2.20%)

**Table 2:** Clinical Characteristics of Veteran Patients by Their Receipt of Chiropractic Care for LBP in the Year Following a Primary Care Visit for Incident Low Back Pain (*N* = 128,377)

Patient clinical characteristics	Chiropractic care on-station		*p*-value
	Yes	No	
*N* (%)	7327 (5.71%)	121,050 (94.29%)	-
High-impact low back diagnoses			
Serious/treatable low back	179 (2.40%)	2612 (2.10%)	0.1040
Radicular low back	1329 (18.10%)	13,918 (11.40%)	< 0.0001
Comorbidities			
Neck pain	2186 (29.80%)	9872 (8.10%)	< 0.0001
Major depressive disorder (MDD)	1335 (18.20%)	21,715 (17.90%)	0.5422
Generalized anxiety disorder (GAD)	293 (3.90%)	3939 (3.20%)	0.0005
Post-traumatic stress disorder (PTSD)	1408 (19.20%)	22,789 (18.80%)	0.4066
Traumatic brain injury (TBI)	111 (1.50%)	1289 (1.00%)	0.0003
Alcohol use disorder (AUD)	348 (4.70%)	6420 (5.30%)	0.0393
Drug use disorder (DUD)	186 (2.50%)	3434 (2.80%)	0.1342
Smoking status			< 0.0001
Never	3193 (43.50%)	48,470 (40.00%)	
Current	2148 (29.30%)	38,881 (32.10%)	
Former	1937 (26.40%)	33,050 (27.30%)	
Missing	49 (6.00%)	649 (5.00%)	
Charlson Comorbidity Index (CCI)			< 0.0001
0	5988 (81.70%)	89,001 (73.50%)	
1	456 (6.20%)	6403 (5.20%)	
2	246 (3.30%)	5574 (4.60%)	
3 +	637 (8.60%)	20,072 (16.50%)	
CCI (continuous) (mean, SD)	0.5 (1.31)	0.89 (1.79)	< 0.0001
Functional Capacity Index (FCI)			0.1682
0	1092 (14.90%)	19,016 (15.70%)	
1	1975 (26.90%)	31,603 (26.10%)	
2	1952 (26.60%)	32,570 (26.90%)	
3 +	2308 (31.40%)	37,861 (31.20%)	
FCI (continuous) (mean, SD)	1.95 (1.40)	1.96 (1.45)	0.6373
Maximum numerical rating score for pain (NRS)—Index Date			< 0.0001
Missing	470 (6.40%)	8583 (7.00%)	
No pain	1428 (19.40%)	23,243 (19.20%)	
Mild	1453 (19.80%)	20,186 (16.60%)	
Moderate	2606 (35.50%)	40,652 (33.50%)	
Severe	1370 (18.60%)	28,386 (23.40%)	
Other nonpharmacologic pain treatment(s)	1837 (25.1%)	36,438 (30.1%)	< 0.0001
Opioid prescribed on index date	98 (1.30%)	2421 (2.00%)	< 0.0001

### Propensity Score-Matched Sample and Proportional Hazards Modeling of Time to Opioid Prescription

The propensity score (PS)-matched sample contained 6477 chiropractic care users and their 19,431 non-user matches (*N* = 25,908). A total of 850 chiropractic users (11.6%) could not be matched and were removed from the matched sample.

**Figure 2:**
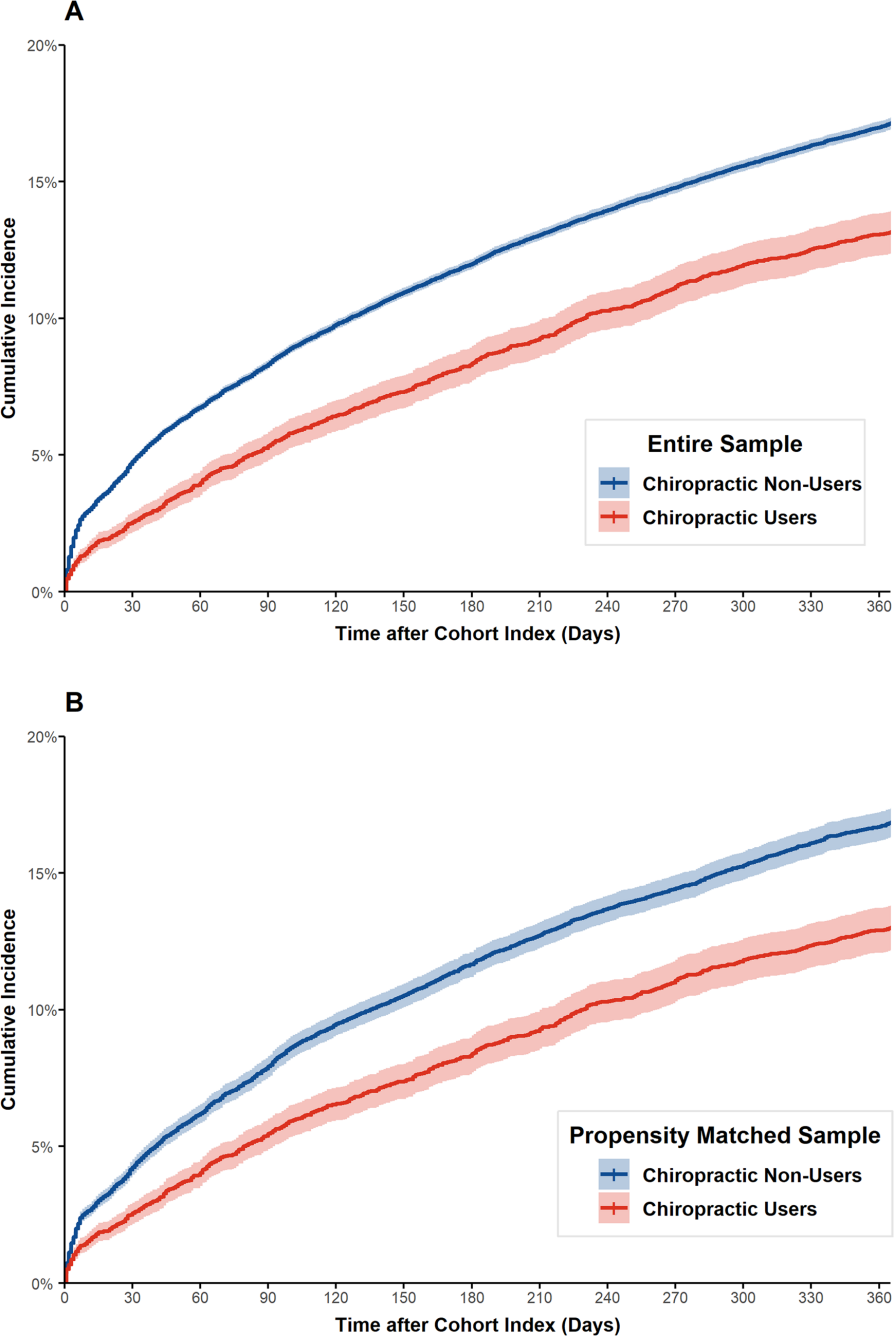
Kaplan–Meier estimates of time to opioid prescription for veterans with low back pain in entire and propensity score-matched sample. A Cumulative incidence of opioid prescription within 365 days of incident LBP visit (entire sample *N* = 128,377). B Cumulative incidence of opioid prescription within 365 days of incident LBP visit (matched sample *N* = 25,908).

Figure 2A presents the cumulative incidence of opioid prescription fills in the entire sample. This was lower in chiropractic care users and remained proportional between users and non-users over time. Across the entire 365 days’ follow-up, the cumulative incidence of opioid prescription fills was 13.2% for chiropractic care users and 17.1% for non-users (number needed to treat, NNT = 26). The unadjusted proportional hazards ratio for opioid prescription for chiropractic users compared to non-users in the full sample was 0.74 (95% CI: 0.70, 0.80).

Figure 2B presents the cumulative incidence of opioid prescription fills in the propensity-matched sample. This was lower in chiropractic care users and remained proportional between users and non-users over time. Across the entire 365 days follow-up, we observe cumulative incidence of opioid prescriptions of 13.0% for chiropractic care users and 16.8% for non-users (NNT = 27). Using proportional hazards modeling with propensity score as a covariate, the hazard ratio for opioid prescription for chiropractic users compared to non-users was 0.77 (95% CI: 0.71–0.83).

## DISCUSSION

Our results add to the existing literature showing an inverse relationship between receipt of chiropractic care and receipt of opioid prescriptions among patients with LBP in private sector^31–37^ and VA^23^ populations. The current work is particularly relevant for VA healthcare since it analyzes a large sample of Veterans actively receiving VA care for LBP conditions, rather than those who may have had an isolated visit with a LBP diagnosis.

By restricting our sample to opioid-naïve patients with incident LBP visits to PCPs, followed by at least 2 additional LBP visits in the next year, we believe we have identified those patients in which PCPs or other providers may be faced with the decision of initiating opioid therapy. By using propensity matching to control for several comorbidities and severity factors, we are confident that the matched sample of chiropractic users are very similar to the non-users, and do not represent more simple and self-limiting cases. Thus, the results of this study suggest that chiropractic care may be an important component of opioid sparing strategies for VA patients with LBP.

Our definition of exposure to chiropractic care (a minimum of 2 VA chiropractic visits within 365 days of the incident PCP visit, with no specification of individual therapeutic procedures used) was established to reflect the current state of chiropractic care delivery in VA. Thus, we feel our chiropractic users received real world VA chiropractic care, but not necessarily optimal VA chiropractic care. Studies aiming to assess dosage^38^ and quality components^39,40^ of VA chiropractic care can inform efforts to improve care delivery to maximize patient outcomes.

Prior studies have reported strong demand among Veterans and PCPs for ready access to chiropractic care and other nonpharmacologic treatment options for LBP and other musculoskeletal pain conditions.^41–43^ VA’s increasing delivery of such therapies is congruent with an evidence-based, patient-centered approach to reduce initiation of opioid prescriptions in key pain conditions such as LBP.^44^ More work is needed to identify the optimal timing, multimodal content, and number of chiropractic visits to obtain the best outcomes for Veterans with LBP conditions. Yet, our current findings have important implications for both Veteran and the US adult general population given the high prevalence of LBP,^45,46^ its associated disability^6^ chronic pain’s association with increased suicide risk,^47^ and the fact LBP has been documented in younger Veterans in more recent years.^26^

This work is limited to assessing VA care provided on-station only and did not include VA-purchased community care. We defined a new episode of LBP care as one starting after a clean period of 18 months without LBP visits, yet we did not assess if subjects may have had LBP symptoms without receiving visits within those 18 months, nor if they had LBP visits prior to the clean period. We defined exposure to chiropractic care as at least two visits within the observation period but did not account for total number or any measure of the content or quality of these visits. We did not assess patient or provider preferences with respect to opioid prescribing and chiropractic care since these are not available in VA EHR data, yet it is possible that these played a role in case outcomes. These preferences and other unknown/unmeasured factors associated with receiving chiropractic care and lower opioids were not captured by the propensity scores. Also, due to limitations in VA prescription medication administrative data, we could not attribute the receipt of an opioid prescription to a specific diagnosis in either group.

Overall, initiation of opioid prescription after incident LBP visits to VA PCPs was significantly lower in Veterans who received subsequent chiropractic visits than those who did not. These results offer important insights for policy and practice as VA continues to refine its healthcare system to connect Veterans to the soonest and best care possible. Presently, chiropractic care is provided in-house at 142 of 173 VA Medical Centers and 170 VA outpatient clinics, and while only 1.3% of VA healthcare users received such care in fiscal year 2022, it is projected that will increase to 2.3% by the end of FY 2027.^48^

Further evaluation of chiropractic care’s substitution effect on other healthcare services, along with economic analyses, would contribute to modeling optimal population access in VA.

## Supplementary Information

Below is the link to the electronic supplementary material.

Supplementary file1 (XLSX 27.3 KB)

Supplementary file2 (DOCX 34 KB)

## Data Availability

The datasets generated and analyzed during this study are not publicly available. To maximize the protection and security of veterans’ data while making these data available to researchers, the US Department of Veterans Affairs (VA) developed the VA Informatics and Computing Infrastructure (VINCI). VA researchers must log onto VINCI via a secure gateway or virtual private network connection (VPN) and use a virtual workspace on VINCI to access and analyze VA data. As per the VA Office of Research and Development Policy, VINCI does not allow the transfer of any patient-level data out of its secure environment without special permission. For questions about data access, contact study lead, AJL (Anthony.lisi@va.gov) or the VA Office of Research and Development (VHACOORDRegulatory@va.gov).
